# Anemia in Pregnant Women and Children Aged 6 to 59 Months Living in Mozambique and Portugal: An Overview of Systematic Reviews

**DOI:** 10.3390/ijerph19084685

**Published:** 2022-04-13

**Authors:** Réka Maulide Cane, José Braz Chidassicua, Luís Varandas, Isabel Craveiro

**Affiliations:** 1 Instituto Nacional de Saúde (INS), Ministério da Saúde (MISAU), Estrada Nacional EN1, Bairro da Vila—Parcela no 3943, Distrito de Marracuene, Marracuene 264, Província de Maputo, Mozambique; chidassicua@gmail.com; 2 Unidade de Ensino e Investigação (UEI) de Saúde Pública Internacional e Bioestatística, Global Health and Tropical Medicine (GHTM), Instituto de Higiene e Medicina Tropical (IHMT), Universidade Nova de Lisboa (UNL), Rua da Junqueira 100, 1349-008 Lisboa, Portugal; 3 Institute for Social and Health Sciences (ISHS), University of South Africa (UNISA), 15 Jean Simonis St., Parow, Cape Town 7530, South Africa; 4 Unidade de Ensino e Investigação (UEI) de Clínica das Doenças Tropicais, Global Health and Tropical Medicine (GHTM), Instituto de Higiene e Medicina Tropical (IHMT), Universidade Nova de Lisboa (UNL), Rua da Junqueira 100, 1349-008 Lisboa, Portugal; varandas@ihmt.unl.pt; 5 Hospital CUF Descobertas, Rua Mário Botas, 1998-018 Lisboa, Portugal; 6 NOVA Medical School, Faculdade de Ciências Médicas Universidade Nova de Lisboa, Campo Mártires da Pátria 130, 1169-056 Lisboa, Portugal

**Keywords:** anemia, pregnant women, children, Mozambique, Portugal

## Abstract

Introduction: Globally, anemia is still a public health issue faced by people in low and high-income countries. This study gives an overview of published scientific articles related to the prevalence, nutritional indicators, and social determinants of anemia in pregnant women and children aged 6 to 59 months living in Mozambique and Portugal. Methods: We performed a review of scientific literature in April 2021, searching for published indexed articles in the last 15 years (2003–2018) in electronic databases. Subsequently, quality assessment, data extraction, and content analysis were performed. Results: We have identified 20 relevant publications. Unsurprisingly, anemia plays a relevant role in disability and life imbalances for these subgroups in Mozambique compared with Portugal. For both countries, data on anemia and iron deficiency in pregnant women and children aged 6 to 59 months old are either outdated or remain unclear. Similarly, studies on social determinants and anemia are also still scarce. Conclusions: A gap of information on anemia, other nutritional indicators, and social determinants in pregnant women and children between 6 and 59 months of age living in Mozambique and Portugal is highly observed. More research is crucial to help achieve the goals established by the Sustainable Development Goals.

## 1. Introduction

Anemia is a condition in which a person’s hemoglobin level is less than normal (below 11 g/dL for pregnant women and children aged 6–59 months) [1,2]. Globally, it is still a public health issue faced by people in both low- and high-income countries and is a concern for adolescent girls, women of reproductive age (particularly during pregnancy), and children in the first years of life [1,3]. It impairs health and well-being in women and increases the risk of maternal and neonatal adverse outcomes [4,5]. Physiologic anemia is the most common cause of anemia in the neonatal period, being more pronounced in preterm infants compared with term infants, accounting that half of the cases are due to iron deficiency. In women, it may develop because of prenatal, perinatal (at delivery), or postpartum hemorrhage [6]. Iron deficiency during pregnancy can persist through lactation, although it may be partially alleviated because of lactational amenorrhea. Poor-quality diets can lead to iron deficiency anemia; on another hand, food and nutrition issues are determinants of iron supplementation and health at all levels, through structural macro-level policies, social conditions, or circumstances of daily life [7]. Worldwide, in 2019, anemia prevalence was 22.8% accounting for a total of 1.74 billion cases. In the same year, the prevalence was highest among children under five years (with a combined prevalence of 39.7%). Globally, 54.1% of anemia cases were mild, 42.5% were moderate, and 3.4% were severe. The regions with the highest-burden were South Asia, Western and Central Sub-Saharan Africa [8]. According to the Global Nutrition Report, anemia affects 613.2 million (32.8% prevalence) adolescent girls and women aged 15 to 49 years, being its prevalence markedly higher in pregnant (35.3 million, 40.1%) than non-pregnant (577.9 million, 32.5%) adolescent girls and women [1]. 

According to data from the last Demographic Health Survey, in Mozambique, about 54.0% of women of reproductive age (15–49 years) and 69.0% of children aged 6–59 months had anemia [9]. It is estimated that in 2015, of a total of 388,345 cases of anemia, diarrhea, fever, and respiratory infections, only 45.2% received adequate health care [10]. Data from the EMPIRE study show that in 2014, the prevalence of anemia in the Portuguese population was 20.4%, and this scenario was classified as a public health problem. Of these cases, the vast majority (84.0%) were unaware of having anemia and only 2.0% of the Portuguese population received some form of treatment for this condition [11]. Estimates for the year 2019 indicated that about 45.8% of pregnant women aged 15–49 years living in Mozambique and 19.4% of pregnant women aged 15–49 years living in Portugal have anemia [12]. Anemia prevalence estimates were 68.2% and 14.3% in children aged 6–59 months living in Mozambique and Portugal, respectively [13]. Among the global nutrition goals of the World Health Organization for 2025, set by the World Health Assembly in 2012 and 2013, set to end malnutrition are a 50.0% reduction in anemia in women of reproductive age and a 30.0% reduction in low birth weight [14,15]. The prevention and treatment of anemia in women of childbearing age can help to reduce the low birth weight, perinatal and maternal mortality, and later diseases throughout life [4].

This study gives an overview of information published related to prevalence, nutritional indicators, and social determinants of anemia in pregnant women and children aged 6 to 59 months living in Mozambique and Portugal. The rationale for choosing these countries was that this review is a substudy of one major study ongoing in two hospitals in Mozambique and Portugal on anemia in pregnant women and children aged 6–59 months. Therefore, it is essential to understand the reality and contrasts by exploring social determinants and possible variations within each country related to anemia in these population subgroups. Based on this, we tried to check the information type published in the last 15 years (2003–2018) on the prevalence of anemia in pregnant women and children aged 6 to 59 months living in Mozambique and Portugal.

Overall, we had primary and secondary objectives of identifying and summarizing the content of publications available on selected open-access databases by type of topics (most or least approached), plus verifying whether nutritional/social determinants were approached or not as a subtopic on anemia’s research linked to Mozambique and Portugal.

## 2. Materials and Methods

### 2.1. Search Strategy and Selection Criteria

This review was guided by the Preferred Reporting Items for Systematic Review and Meta-Analysis (PRISMA) guidelines (Supplementary File S8). We performed a review of scientific articles in April 2021, searching for published indexed articles about anemia in pregnant women and children aged 6 to 59 months living in Mozambique or Portugal in the following electronic databases: PubMed, GoogleScholar, ScienceDirect, Cochrane, Scielo, and Lilacs, using Boolean operators (AND, OR). The keywords used were as follows: maternal anemia, children from 6 to 59 months of age, iron deficiency, nutritional indicators, social determinants, ‘anemia materna’, ‘crianças dos seis aos 59 meses de idade’, ‘deficiencia de ferro’, ‘indicadores nutricionais’, ‘niños de seis a 59 meses de edad’, ‘indicadores nutricionales’ and ‘determinantes sociales’. No MeSH descriptors (terms) were used. Further information regarding the search strategy is included in Table 1 and Supplementary File S4.

The selection of papers was restricted to articles with results of studies focused on anemia in pregnant women and children aged 6 to 59 months living in Mozambique or Portugal published between the years 2003 and 2018, in English, Portuguese or Spanish languages and that were available on the selected electronic databases. This review was limited to 15 years (2003–2018) as we were primarily interested in looking at the scientific evidence published in a similar time that major demographic health national surveys and population-based studies involving anemia data were held in Mozambique and Portugal [9,16,17,18,19]. Therefore, we expected to help to refine the development of subsequent analysis involving the mentioned surveys and anemia data. We analyzed the prevalence of anemia according to the World Health Organization’s criteria [20]. Individual studies, technical reports, theses, technical specifications and standards, noncommercial translations, technical and commercial documentation, and official documents not published were excluded from this review. Articles without abstract and where the full text could not be obtained, as well as articles with duplicate data (data from the same study published in different journals) were also excluded. Table 2 summarizes the eligibility criteria used for the inclusion and exclusion of articles.

Two investigators (RMC and JBC) performed a double-blinded independent screening of title/abstract, selection, and full-text review. The final selection process was blinded and independent using the feature “Blind-On” available on Rayyan—intelligent systematic review software (http://rayyan.qcri.org, accessed on 29 April 2021) [21], which helps to reduce the influence by the decisions of the researchers during the article selection stage (hiding individual authors’ decisions about included studies), thus removing a significant possible source of bias [21,22]. The verification of duplicates was performed and articles without abstract and/or without full text, which did not address anemia, which did not include the countries of interest (Mozambique and/or Portugal), which did not address the target groups, and written in languages (other than Portuguese, English or Spanish) were excluded. Findings were subsequently verified and synthesized. Discussion and consensus were used to solve any emerged discrepancies between the two investigators (RMC and JBC).

### 2.2. Quality Assessment and Data Extraction

To assess the quality of the publications, 22 criteria of the STROBE -Strengthening the Reporting of Observational Studies in Epidemiology checklists were applied [23,24,25]. These criteria involve a series of characteristics that relate to the title, abstract, introduction, statistical methods, bias, results, and discussion sections of the articles [24] and were used independently by two members of the team (RMC and JB) for the evaluation. An excel spreadsheet was elaborated for the verification and calculation of the percentage of methodological quality (Supplementary Files S1 and S2). We also performed the critical appraisal of the publications using the ‘AMSTAR 2′—A Measurement Tool to Assess systematic Reviews software (https://amstar.ca/index.php, accessed on 7 May 2021) [26,27].

With the completion of the quality assessment, we performed (RMC and JBC) to the extraction of relevant data, the content analysis, and the summarization of the information. The extracted data included the following information: author(s), year of publication, mentions of Mozambique and/or Portugal, target groups, Mozambique or Portugal specific findings, and period of analysis/analysis performed (Table 2 and Supplementary File S3). The description of relevant findings by thematic categories was also included (Supplementary File S6). We contacted the corresponding author in cases we were unable to access the article’s Supplementary Files. 

## 3. Results

**Figure 1:**
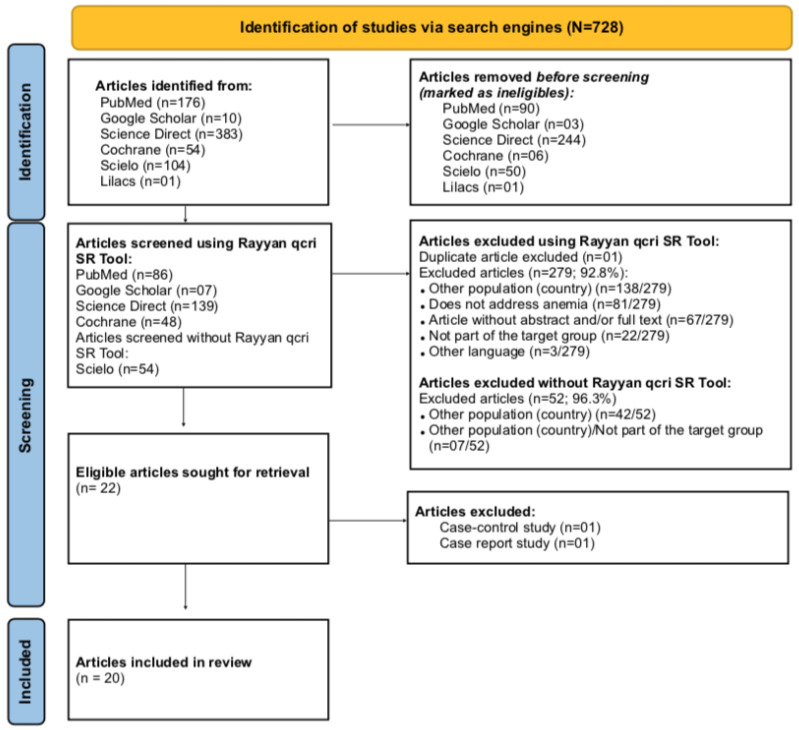
PRISMA flow chart describing selection of articles for review on anemia in pregnant women and children aged 6 to 59 months living in Mozambique and Portugal.

We identified a total of 728 articles using our search criteria (Figure 1). Subsequently, considering the period of publication (2003–2018) and the country of the study, 334 articles were picked for the final selection stage. Of these, 280 articles were obtained from PubMed, Google Scholar, ScienceDirect, Cochrane electronic databases, exported and selected using the Rayyan, whereas the other 54 articles were obtained from the Scielo database and were selected without Rayyan due to the incompatibility of the file formats. In the final selection process, the verification of duplicates was performed, and articles were excluded using the exclusion criteria previously mentioned. A total of 20 articles were considered eligible (2.7%; 20/728) (Figure 1). These 20 potentially relevant articles were published between 2009 and 2018; 45.0% (9/20) mentioned Mozambique [28,29,30,31,32,33,34,35,36]; 15.0% (3/20) mentioned Portugal [37,38,39] and 40.0% (8/20) mentioned both countries [40,41,42,43,44,45,46,47] (Supplementary File S3).

**Figure 2:**
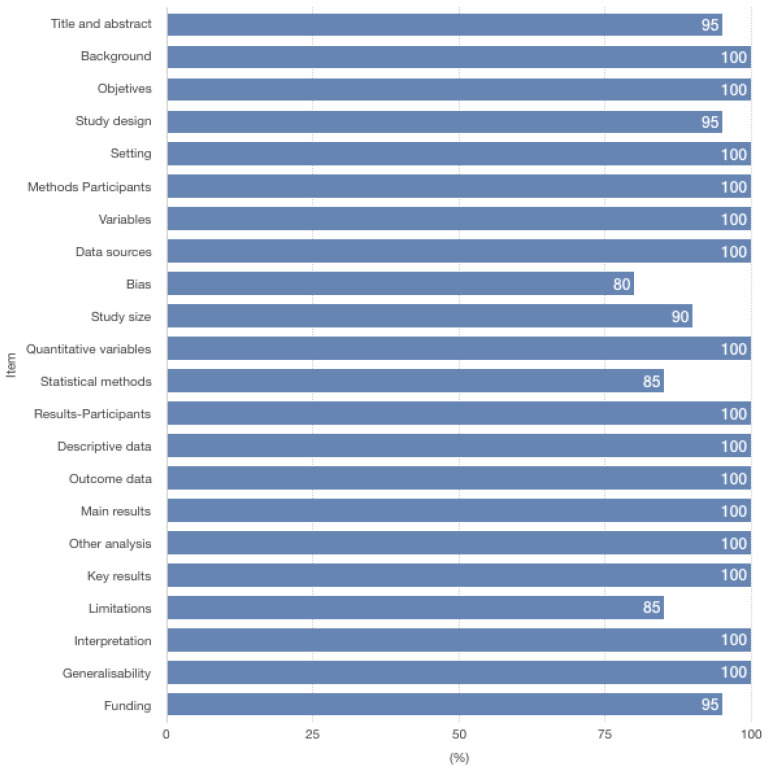
Methodological quality evaluation of articles included.

Most of the publications were on the infancy and childhood subgroup (65.0%; 13/20) and studied mainly issues centered on anemia/iron deficiency and years of life lost, disability-adjusted life years [30,32,33,34,35,36,40,42,43,44,46,47] (Supplementary File S3). Regarding the quality assessment, we verified that, in general, most of the checklist items were applied (95.0–100%). Few studies did not apply/apply correctly items related to bias (80%), limitations (85%) and statistical methods (85%). Overall, the articles selected had a good methodological quality (Figure 2 and Supplementary File S2).

**Figure 3:**
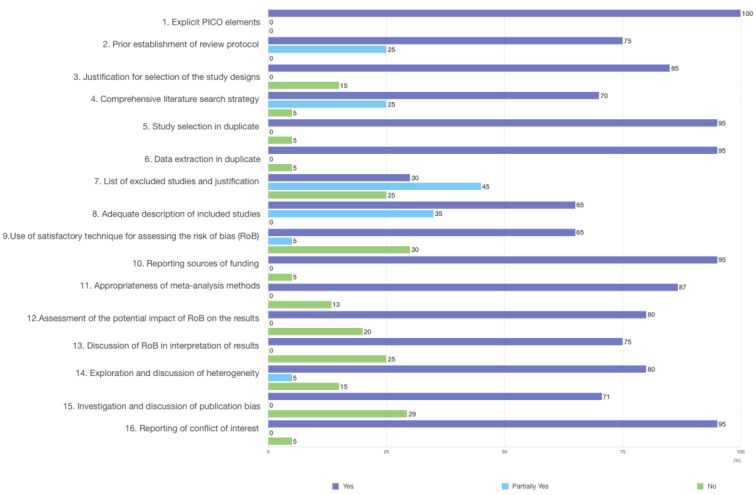
Quality assessment of articles included, by AMSTAR 2 critical domains.

Regarding the AMSTAR 2 critical domains, 60.0% performed a comprehensive literature search, 95.0% performed data selection and extraction in duplicate, 65.0% used satisfactory techniques for assessing the risk of bias, 71.0% discussed or investigated publication bias, and 95.0% reported conflicts of interest (Figure 3; Supplementary Files S2.1 and S2.2). 

We also determined the percentage of studies that approached the following topics of interest: maternal anemia/anaemia, childhood anemia/anaemia, prevalence of anemia/anaemia, maternal nutritional indicators (folic acid supplementation and powder micronutrients supplementation/fortification), infant and young infant nutritional indicators (breastfeeding, exclusive breastfeeding, infant formula), and social determinants (age, gender, years of schooling, wealth index). Iron deficiency anemia (65.0%; 13/20) [31,32,33,34,35,37,40,41,42,43,44,46,47] and prevalence of anemia (35.0%; 7/20) [30,33,34,37,40,42,43] were the most mentioned, whereas maternal and infant anemia were the least mentioned (10.0%; 2/20) [28,30] (Supplementary File S3). Regarding nutritional indicators, few articles mentioned maternal nutritional indicators which were related to supplementation with folic acid during pregnancy (10.0%; 2/20) [32,34] and the use of micronutrients for home fortification (5.0%; 1/20) [35] (Supplementary File S3). Breastfeeding was the only infant and young infant nutritional indicator mentioned across the selected publications (5.0%; 1/20) [33]; other indicators such as exclusive breastfeeding or infant formula were not mentioned. Age was the social determinant more studied (65.0%; 13/20) [28,31,32,33,39,40,41,42,43,44,45,46,47] and the least studied were linked to years of schooling (5.0%; 1/20) [43] and wealth index (5.0%; 1/20) [31] (Supplementary File S3).

We performed a content analysis to identify the type of information published and specific key findings to Mozambique and Portugal. Table 3, Supplementary Files S3 and S6 summarize the general and specific country findings of the 20 publications.

### 3.1. Anemia and Iron Deficiency Anemia (IDA) Prevalence

Global and regional anemia and iron deficiency anemia prevalence data were reported in five studies, but only two studies reported Mozambique and Portugal-specific data [30,33,40,42,43]. In 2013, the prevalence of iron deficiency cases (by thousands) was of about 5193.9 (4985.7–5442.7) and of about 1205.7 (1180.2 to 1227.5), in Mozambique and Portugal, respectively [40]. The absence of postpartum anemia in pregnant women with vaginal deliveries who were admitted in labor was also reported in one study trial conducted in Portugal during the years 2006 and 2007 [38]. A review reported that in 2005, the prevalence of anemia was severe (≥40.0%) among Mozambican children of preschool age (0–5 years) [30]. Nonetheless, for both countries, data on anemia and iron deficiency in pregnant women and children aged 6 to 59 months old are either outdated or remain unclear as the findings hereby reported were not disaggregated into these specific subgroups.

### 3.2. Years Lived with Disability (YLDs) and Disability-Adjusted Life-Years (DALYs)

Eight studies provided data on anemia, iron deficiency, years lived with disability (YLDs), and disability-adjusted life-years (DALYs) [39,40,41,42,43,44,45,46]. It was documented that iron deficiency anemia was among the ten causes of years lived with a disability (YLD) during the year 2013 in Mozambique, but not for Portugal [40]. For the same year, Mozambique’s YLDs iron deficiency anemia was of about 199.3 (133.8 to 290.1) and of 29.4 (19.2 to 43.1) in Portugal. Between 2015 to 2016 anemia was among the top ten leading causes of disability in Mozambique (ratio of observed YLD to YLD on basis of the social demographic index was 0.71 and 0.98, respectively) but not in Portugal. For the same period, iron deficiency anemia was also among the leading Level 4 causes of age-standardized YLD rates for females in Mozambique but not in Portugal [42,43,44]. Concerning DALYs, one study reported that in 2013 children undernutrition and iron deficiency were ranked as 2nd and 9th risk factors among those ten risk factors of attributable DALYs for males and females in Mozambique. Iron deficiency wasn’t among the ten leading risk factors in terms of DALYs neither in Mozambique nor Portugal, for both sexes combined [41]. Nonetheless, one study reported that in the same year, iron deficiency was one of the top ten causes of DALYs for adolescent girls aged 10–19 years old [37]. Overall, anemia and iron deficiency anemia play a crucial role among children and adolescent girls living in Mozambique-a country still facing a high burden of malnutrition [32].

### 3.3. Malaria and HIV/AIDS

Two study trials that included 5469 pregnant women conducted between 1987–2013 in Mozambique, showed that mefloquine was more efficacious than sulfadoxine-pyrimethamine in HIV-uninfected women or daily cotrimoxazole prophylaxis in HIV-infected pregnant women for prevention of malaria infection and was associated with lower risk of maternal anemia (RR 0.84, 95% CI 0.76 to 0.94), no adverse effects on pregnancy outcomes (stillbirths and abortions), and no effects on low birth weight and prematurity. The high proportion of mefloquine-related adverse events constitutes an important barrier to its effectiveness for malaria preventive treatment in pregnant women [28]. Two other trials carried between 2004–2008 in Mozambique found a large effect in reducing the risk of cord blood anemia (RR 0.49, 95% CI 0.30 to 0.80), and increase in mean cord packed cell volume (MD 1.01%, 95% CI 0.05 to 1.97) [29]. Nonetheless, the possible effects or benefits of routine malaria chemoprevention, hookworm and HIV/AIDS prevention and treatment strategies and hookworms on reducing the risk of maternal anemia and negative pregnancy outcomes are still not well documented, in particular in provinces of Mozambique with the highest burden of malnutrition and food insecurity. Unsurprisingly, for Portugal comparatively to Mozambique, which has been facing a burden of malaria and HIV/AIDS for several years, data remain unclear.

## 4. Discussion

In this overview, we have identified 20 relevant publications. Regarding specific results on anemia in both countries, few were linked to anemia and iron deficiency prevalence and those related to the causes of disability and life imbalances were commonly presented [30,37,40,41,42,43,44,45]. 

In 2013, Mozambique showed slightly lower iron deficiency prevalence rates and YLDs iron deficiency anemia values compared to neighboring countries such as Tanzania ((with a prevalence of iron deficiency cases of 55,599,526.2 (9365.7–9731.6); and the YLDs iron deficiency anemia values of 346.2 (229.1–498.1)). Likewise, the same data was valid for Portugal in comparison with the neighboring country, Spain ((with a prevalence of iron deficiency of 5907.3 (5756.9–6026.4); and the YLDs due to iron deficiency anemia values of about 145.2 (96.2–213.4)) [40]. Concerning DALYs, in 2013, similarly to Mozambique children’s undernutrition was ranked also as 2nd risk factor for other countries of Eastern Sub-Saharan Africa such as Djibouti, Kenya, Malawi, Tanzania, Uganda, and Zambia. Nonetheless, differently from Mozambique, iron deficiency was ranked not as the 9th risk factor but the 7th for Somalia and as the 10th for Burundi, Comoros, Djibouti, Malawi, and Tanzania [41]. Undoubtedly, anemia and iron deficiency anemia continue persisting as an unsolved risk factor/cause of DALYs throughout the past years in Mozambique and possibly overshadowing the in-country fight against maternal and child morbidity and mortality.

Evidence indicates that anemia plays a relevant role in disability and life imbalances in pregnant women and children under five years of age in Mozambique compared with Portugal [40,42,43]. These findings are consistent with previous studies in Portugal that showed a low prevalence of maternal anemia in the first half of pregnancy. However, careful attention needs to be paid as these studies were conducted with Portuguese women with the 20th week of pregnancy, whereas anemia prevalence often tends usually to be higher in late pregnancy, in women without iron supplementation [48]. Our findings related to Mozambique agree well with previous reports that suggest that we are faced with a population of mothers with low iron stores, conditioning the hemoglobin values of infants in the first months of life [49]. Adding to this are many contextual cultural and socio-economic factors that may contribute to high levels of anemia throughout infancy in Mozambican children.

A growing body of literature showed that adolescents are at risk of iron deficiency because of their high iron requirements during the growth spurt period, particularly in girls for whom the start of menstruation leads to iron losses [50,51,52,53]. Concerning adolescent women, iron deficiency anemia is still a public health problem in both countries, since the data indicate that it is a risk factor for disadjusted life years [37,41]. The results found in Portuguese adolescent girls, also coincide with those reported in the last statistics of Portugal that showed prevalence rates of 9.0% in adolescent girls aged 12–15 years and 16.0% in adolescent girls aged 16–19 years [54]. Reports from previous years from the HELENA Study showed that the overall proportion of iron depletion among adolescents was 17.6%, being higher in girls compared to boys. By geographical location, rates were higher in Eastern Europe (Pecs, 23%) followed by Northern Europe (Stockholm, 19.0%), Western Europe (17.0% in Ghent and 19.0% in Lille), Central Europe (16.0% in Dortmund and 19.0% in Vienna) and 15.0% in Southern Europe (14.0% in Athens, 17.0% in Heraklion, 19.0% in Rome and 10.0% in Zaragoza) [51]. Other authors reported a prevalence of anemia of 2.6% in Portuguese adolescents living in the city of Porto, being these rates higher in girls (4.1%) compared to boys (1.0%) [50,55]. Nonetheless, these results only allow for statistical comparison of differences in iron status between selected European cities but not between European regions [51]. On the other hand, our findings are similar to those found in Spain, which shows that 15.0% of Spanish adolescents had iron deficiency anemia in a similar period, thus pointing more specifically to iron deficiency anemia as a clinical challenge in the daily practice of medicine at all levels of care [56]. There is evidence to suggest that dietary iron intake may be poor because of inadequate intake during adolescence or due to poor iron intake since infancy. Other factors such as a change in dietary habits by peer influence, eating disorders (refusal to eat, excessive weight-loss diets, and skipping meals), dependence on food that can be prepared rapidly and simply (fast food) can also play a crucial role on iron deficiency [50]. Our findings suggest the need for specific attention to adequate dietary intake from infancy up to adolescence particularly to adolescent girls ensuring that their dietary iron intake is adequate to their requirements, agreeing with those results previously reported by De Andrade et al. [50]. Findings here reported related to anemia in Mozambican preschoolers under five years of age are also of huge concern, as other studies conducted in Sub-Saharan Africa showed that anemia persists through later ages of life, being also a burden in elder preschoolers aged 7–15 years [30,57]. 

The global burden of diseases in 2015 and 2016 classifies Mozambique as a low social demographic index country (SDI) and Portugal as a high-middle social demographic index country (SDI) [42,43,46]. In the year 2016, iron deficiency anemia was one of the leading causes of YLDs in low-middle-SDI and low-SDI quintiles. Pooled analysis that included among other African countries, neighbor-countries of Mozambique with a similar SDI (such as Tanzania, Eswatini, and Madagascar), showed that the risk of anemia among women living in the lowest wealth quintiles was 25.0% higher than among those in the highest wealth quintile. Women with no education were more likely to be anemic than were those with greater than secondary education. Patterning of anemia by socioeconomic status was also noted for children: a child living in a household in the lowest wealth quintile was 21.0% more likely to be anemic than were those in the highest wealth quintile [30]. Conditional on demographic and socioeconomic factors, the mother’s anemia status was among the strongest predictors of anemia in children [30,34]. Our review shows that age and gender were the social determinants more analyzed comparatively to indicators such as the years of schooling or wealth index [37,40,42,43]. Previous studies highlighted the relevance of social determinants analysis to better understand the causal association of social determinants and the occurrence of anemia [58]. Despite the relevance of this issue has been studied in other countries hereby mentioned, our review shows that, limited research evidence on anemia’s social and structural determinants prevails for Mozambique and Portugal.

A gap of information on anemia, other nutritional indicators (in addition to iron deficiency), and social determinants in pregnant women and children between 6 and 59 months of age living in Mozambique and Portugal are highly observed in this study overview. Little literature related to anemia; our focus subgroup was also found at Index Medical Portuguese Journals—a Portuguese national database with non-open access. Anemia prevalence among Portuguese pregnant women and Mozambican children under 59 months old was a topic approached in some of the publications found, nonetheless, the majority of studies had its focus on supplementation with iron during pregnancy, preschoolers’ food habits, and case studies interlinked with anemia during infancy [48,49,59,60,61,62,63,64]. More research is required to gather scientific-based evidence that can contribute to improving strategies that allow us to achieve the goals established by the World Assembly of WHO of reducing anemia in women of reproductive age, low birth weight, and under-five mortality. Undoubtedly efforts to attain these goals are interlinked to achieving the following Sustainable Development Goals (SDGs): “2.2 End all forms of malnutrition”, “3.1. Reduce global maternal mortality ratio to less than 70 per 100 000 live births by 2030” and “3.2. End preventable deaths of newborns and children under five years of age, with all countries aiming to reduce neonatal mortality to at least as low as 12 per 1000 live births and under-5 mortality to at least as low as 25 per 1000 live births, by 2030” [65]. 

An opportunity acknowledged by the Universal Health Coverage (UHC) 2030′s platform is that the inclusion of universal health coverage in the SDGs can be a coherent approach to health, allowing the acceleration of equitable and sustainable progress toward universal health coverage and health systems strengthening at global and country levels [66]. Even though both countries which were the focus of our study present huge contextual, geographic, and socioeconomic differences, Mozambique despite some progress (made in previous years) had a remarkably low UHC service coverage index and life expectancy (of 46.0; 61 years) compared with Portugal (of 82.0; 80 years) by the year 2017. External health expenditure was also strikingly higher in Mozambique (62.92) compared with Portugal (0.09) by the year 2018 [66,67]. With this in mind, the best performances in health indicators are clearly observed for Portugal compared with Mozambique where major health (triple burden diseases, infectious illnesses), lack of water, lack of good sanitation, lack of access to health facilities problems, as well as food insecurity still prevails [68]. Nevertheless, Costa et al. showed that throughout Eastern and Southern European countries, including Portugal, population health inequalities prevail across metropolitan areas, generally, with municipalities presenting worse health determinants value scores. Despite geographic disparities in the distribution of value scores of health outcomes between municipalities may not be as evident as expected in Portugal, more specifically in Lisbon, health inequalities still prevail and need to be tackled [69]. As stated by Malta et al. [68], Mozambique and Portugal are both Community of Portuguese Language Countries (CPLP), and the strengthening of network collaborations focused on research, and more specifically on anemia can be of added value to give more visibility to this health issue and to improve health policies and to reinforce strategies aimed to reduce anemia between pregnant women and children under five years living in Mozambique and Portugal.

## 5. Conclusions

This review highlighted the research gap on anemia, iron deficiency among pregnant women and children living in Mozambique and Portugal. 

Anemia and iron deficiency anemia continue persisting as an unsolved risk factor/cause of DALYs especially for Mozambique- highly burdened by major health burden and food insecurity challenges. For both countries, data on anemia and iron deficiency in pregnant women and children aged 6 to 59 months old reported in the studies are either outdated or remain unclear as most of the findings were not disaggregated into these specific subgroups. 

There is a lack of information on the effects of malaria chemoprevention, HIV/AIDS prevention and treatment strategies, micronutrient supplementation, iron correction treatment on women of reproductive age, pregnant women and children under 59 months living in these countries, as well, on the influence of social-structural determinants into the risk of anemia due to inequalities, especially in the current context of COVID-19 pandemic. 

The strengthening of international and/or Community of Portuguese Language Countries (CPLP) network collaborations for innovative research on anemia and iron deficiency among this specific population group is needed, as well as the increase in awareness of anemia as impairment for life quality and a risk factor for maternal and child morbidity and mortality. Better resource-focused strategies for nutritional education, prevention, and treatment of anemia and iron deficiency can also be drawn by using more realistic and updated socio-contextual in-country-based evidence.

## 6. Limitations

The reporting of results of studies conducted in Mozambique and Portugal may be underestimated. As it is possible that some studies may have been conducted after the period set for this review or may be found in other databases (private, difficult to access by common users, or not included in this overview).

Our review was limited to 15 years (2003–2018) due to reasons previously stated. Once we finished our review, and despite being out of our primary study focus conduct any further evaluation of its content, we performed an update search and identified only five potentially relevant studies (Supplementary File S6) [70,71,72,73,74]. Thus, we observed that publications on this specific issue are still scarce for both countries. Language may also be a source of bias as most of the journals indexed in major databases are often published in the English language. Therefore, the actual number of studies might not be representative and be only a subset of studies that are being performed in these two Portuguese Language countries. We tried to overcome this limitation by broadening our search using additionally to the English language the Portuguese and Spanish languages.

## Figures and Tables

**Table 1:** Search terms and operators used for the identification of publications in the electronic databases.

Electronic Databases	Terms and Operators
**PubMed**	MATERNAL ANEMIA OR MATERNAL ANAEMIA OR ANEMIA MATERNA AND CHILDREN FROM SIX TO 59 MONTHS OF AGE OR CRIANCAS DOS SEIS AOS 59 MESES DE IDADE OR NIÑOS DE SEIS A 59 MESES DE EDAD AND IRON DEFICIENCY OR DEFICIENCIA DE FERRO OR DEFICIENCIA DE HIERRO AND NUTRITIONAL INDICATORS OR INDICADORES NUTRICIONAIS OR INDICADORES NUTRICIONALES AND SOCIAL DETERMINANTS OR DETERMINANTES SOCIAIS OR DETERMINANTES SOCIALES
**GoogleScholar**	MATERNAL ANEMIA OR MATERNAL ANAEMIA OR ANEMIA MATERNA AND CHILDREN FROM SIX TO 59 MONTHS OF AGE OR CRIANCAS DOS SEIS AOS 59 MESES DE IDADE OR NIÑOS DE SEIS A 59 MESES DE EDAD AND IRON DEFICIENCY OR DEFICIENCY DE FERRO OR DEFICIENCIA DE HIERRO AND NUTRITIONAL INDICATORS OR INDICADORES NUTRICIONAIS OR INDICADORES NUTRICIONALES AND SOCIAL DETERMINANTS OR DETERMINANTES SOCIAIS OR DETERMINANTES SOCIALES
**Science Direct**	MATERNAL ANEMIA (OR) MATERNAL ANAEMIA (AND) CHILDREN FROM SIX TO 59 MONTHS OF AGE (AND) IRON DEFICIENCY ((AND) NUTRITIONAL INDICATORS (AND) SOCIAL DETERMINANTS
**Cochrane**	MATERNAL ANEMIA OR MATERNAL ANAEMIA OR ANEMIA MATERNA AND CHILDREN FROM SIX TO 59 MONTHS OF AGE OR CRIANCAS DOS SEIS AOS 59 MESES DE IDADE OR NIÑOS DE SEIS A 59 MESES DE EDAD AND IRON DEFICIENCY OR DEFICIENCIA DE FERRO OR DEFICIENCIA DE HIERRO AND NUTRITIONAL INDICATORS OR INDICADORES NUTRICIONAIS OR INDICADORES NUTRICIONALES AND SOCIAL DETERMINANTS OR DETERMINANTES SOCIAIS OR DETERMINANTES SOCIALES
**Scielo**	MATERNAL ANEMIA (AND) CHILDREN FROM SIX TO 59 MONTHS OF AGE (AND) IRON DEFICIENCY
**Lilacs**	(MATERNAL ANEMIA) OR (MATERNAL ANAEMIA) OR (ANEMIA MATERNA) AND (CHILDREN FROM SIX TO 59 MONTHS OF AGE) OR (NIÑOS DE SEIS A 59 MESES DE EDAD) AND (DEFICIENCIA DE HIERRO) OR (IRON DEFICIENCY) AND (NUTRITIONAL INDICATORS) OR (INDICADORES NUTRICIONALES)

Notes: Languages used for the search include Portuguese, English and, Spanish.

**Table 2:** Eligibility criteria used for inclusion and exclusion of articles.

Inclusion Criteria	Exclusion Criteria
Anemia; Pregnant women and children aged 6 to 59 months; Mozambique and/or Portugal; Reviews or systematic reviews (secondary research); Articles published between 1 January 2003 up to 31 December 2018; Articles published in English, Spanish or Portuguese languages.	Did not address anemia; Without abstract and/or without full text; Did not include the countries of interest (Mozambique and/or Portugal); Did not address the target groups; Individual studies (primary research); Written in languages other than Portuguese, English or Spanish.

**Table 3:** Description of the articles included, by year of publication, country’s mention, target group, country’s specific findings, period of study and analysis performed.

Reference No.	Author	Year of Publication	Mentions on Mozambique/Portugal	Target Group	Mozambique/Portugal Specific Findings	Period of Analysis/Analysis Performed	General Findings Related to Anemia
[28]	González et al.	2018	Mozambique	Pregnant women (trials, antenatal care clinic settings)	Mefloquine probably results in fewer women anemic at delivery (when compared with sulfadoxine-pyrimethamine);Mefloquine plus cotrimoxazole probably results in little or no difference in maternal anemia cases at delivery	2010–2013; Systematic review: Dichotomous outcomes were compared using risk ratios (RRs), count outcomes as incidence rate ratios (IRRs), and continuous outcomes using mean differences (MDs).	When compared with sulfadoxine-pyrimethamine, mefloquine decreased maternal anemia at delivery (RR 0.84, 95% CI 0.76 to 0.94; 5469 participants, 2 studies; moderate-certainty evidence). When compared with cotrimoxazole, there was no probably effect on maternal anemia at delivery (RR 0.94, 95% CI 0.73 to 1.20; 1197 participants, 2 studies; moderate-certainty evidence).
[29]	Radeva-Petrova et al.	2014	Mozambique	Pregnant women (Trials, conducted between 1957 and 2008)	Of the two most recent trials, both large, and both administering two doses of sulfadoxine-pyrimethamine (SP), one trial from Mozambique demonstrated a benefit with chemoprevention(ofr maternal outcomes); One trial in Mozambique found a large effect in reducing the risk of cord blood anemia (RR 0.49, 95% CI 0.30 to 0.80; one trial, 870 participants).	Systematic review: Dichotomous outcomes were compared using risk ratios (RR), and continuous outcomes using mean differences (MDs).	“For women in their first or second pregnancy, malaria chemoprevention reduces the risk of moderate to severe anemia by around 40% (RR 0.60, 95% CI 0.47 to 0.75; three trials, 2503 participants, high quality evidence), and the risk of any anemia by around 17% (RR 0.83, 95% CI 0.74 to 0.93; five trials,, 3662 participants, high quality evidence).”
[30]	Balarajan et al.	2011	Mozambique	Children of preschool age (0–5 years) and Pregnant women	Anemia prevalence in mozambican children of preschool age (0–5 years) was severe (≥40.0%)	1993–2005; Revision, including discussion of the multifactorial causes of anemia and the co-occurrence of multiple risk factors in different populations and identify potential barriers to effective anemia prevention and control.	“Africa and Asia were the most heavily affected regions, with Africa having the highest prevalence of anemia, and Asia bearing the greater absolute burden.”
[31]	Arsenault et al.	2018	Mozambique	Pregnant women, Women of reproductive age (15–49 years) who had at least one live birth in the past 2 years (MICS) or 5 years (DHS)	No specific country findings for anemia were presented; In Mozambique, more than 90% of women accessed skilled antenatal care but less than 60% reported the three services (quality antenatal care). Mozambique had high levels of coverage but low and inequitable levels of quality	The availability of potential indicators of ante-natal care quality in household surveys was assessed.	“Quality antenatal care involves the provision of respectful, evidence-based care including appropriate patient assessments such appropriate preventive and curative treatments (eg, iron supplementation); and patient counseling and education.”
[32]	Bhutta et al.	2013	Mozambique	Pregnant women and Children	No specific country findings for anemia were presented	Comprehensive review: the potential effect of delivery of nutrition specific interventions on lives saved in the 34 countries with 90% of the global burden of stunted children was modeled	Folic acid supplementation during pregnancy improved means birth weight, with a 79% reduction in the incidence of megaloblastic anemia; and iron supplementation to women during pregnancy contributed to 70% reduction in anemia at term, a 67% reduction in iron deficiency anemia (IDA), and 19% reduction in the incidence of low birthweight.
[33]	Black et al.	2013	Mozambique	Children < 5 years and Pregnant women	No specific country findings for anemia were presented. Prevalence of stunting (HAZ < –2 Z scores below median) and overweight (BAZ > 2 Z scores above median) for highest and lowest wealth quintiles for Mozambique (MICS 2006) were presented	2013; Proportion of pregnant women with anemia whose blood hemoglobin would increase to at least 110 g/L was calculated. The proportion of severe anemia that would increase to at least 70 g/L was also calculated.	Iron deficiency contribute substantially to maternal deaths and maternal iron deficiency is associated with babies with low weight (<2500 g) at birth; anaemia (haemoglobin <110 g/L), which might be attributable to low consumption or absorption in the diet or to blood loss, such as from intestinal worms, is highly prevalent during pregnancy. In Africa, the prevalence of iron deficiency anaemia (haemoglobin <110 g/L) was of 20.2% (18.6–21.7) and 20.3% (18.3–22.4) in children < 5 years and pregnant women, respectively. In Europe, the prevalence of iron deficiency anaemia (haemoglobin < 110 g/L) was of 12.1% (7.8–16.2) and 16.2% (12.6–19.7) in children < 5 years and pregnant women, respectively.
[34]	Duncan, Burris	2009	Mozambique	Pregnant women and infants	No specific country findings for anemia were presented.	Overview of the state of the world’s children from the late 1970s until the year 2008.	“Complications from a teen pregnancy are numerous and include high prevalence of anemia. Universal supplementation of calcium, iron, and folic acid during pregnancy can prevent almost one-fourth of all maternal deaths.”
[35]	Gaffey et al.	2015	Mozambique	Children aged < 12 years	No specific country findings for anemia were presented	1990–2015; Revision of global and regional progress towards Millennium Development Goals (MDGs) 4 and 5 with respect to their indicators	Intermittent iron supplementation in children aged < 12 years is associated with a 49% lower risk of anemia and a 76% lower risk of iron deficiency. The evidence on the use of micronutrient powders (MNPs) for home fortification suggests a 33% reduction in anemia, a 57% reduction in iron deficiency anemia.
[36]	Ruel et al.	2018	Mozambique	Children (0–3 years for Mozambique’s specific studies)	No specific country findings for anemia were presented	Revision of the nutrition impacts of agricultural programs with new empirical evidence published from 2014 onwards.	-
[37]	Langer et al.	2015	Portugal	Adolescent girls (10–19 years)	No specific country findings for anemia were presented; In 2013, iron deficiency anemia was among the top ten causes of disadjusted life years (DALYs) for 10–19 years-old adolescents girls (DALYs = 5.1).	Analysis of the major economic, environmental, social, political, demographic, and epidemiological transitions happening worldwide, their implications on the health system, and their effects on women and health;	Iron deficiency anemia is among the most important risk factors for mortality for girls aged 15–19 years, accounting for a substantial portion of DALYs through its contribution to cognitive impairment, susceptibility to infection, and limited work capacity. It is also a major factor in more than 115 000 maternal deaths and 591,000 perinatal deaths worldwide every year.
[38]	Diaz et al.	2018	Portugal	Pregnant women (more specifically women with vaginal deliveries who were admitted in labour; Trial conducted between 2006 and 2007)	There was not report on postpartum anemia (defined as Hb lower than 9 mg/dL).	Systematic review	“Depending on the rate of blood loss and other factors, such as pre-existing anemia, untreated postpartum hemorrhage can lead to hypovolemic shock, multi-organ dysfunction, and maternal death, within two to six hours. The cluster-randomized trial (Zhang 2010), was conducted between 2006 and 2007 and included 25,381 women with vaginal deliveries who were admitted in labour to the 78 maternity units participating in the study (included 05 maternity units in Portugal).”
[39]	De-Regil et al.	2015	Portugal	Cohort study of births (1988–1988) in 10 countries (including Portugal)	No specific country findings for anemia were presented, as the study design of the research was out of the scope of the review.	Systematic review	-
[40]	Vos Theo et al.	2015	Mozambique and Portugal	Childhood and Individuals aged 15–49 years and 50–69 years	Iron deficiency anemia was among the ten causes of years lived with disability in Mozambique (in 2013). Anemia wasn’t among the 10 leading causes of disability in Portugal	1990–2013; Estimations of anemia prevalence and distribution of mild, moderate, and severe anemia by cause; Disaggregation of marginal estimates of anemia severity and cause; years lived with disability(YLD) computations	“By 2013, 49·2% of individuals had mild anaemia, 46·9% had moderate anaemia, and 3·9% had severe anaemia. Iron deficiency anaemia accounted for 62·6% of all cases and 31·5% of mild, 28·7% of moderate, and 2·4% of severe anaemia.”
[40]	Wang et al.	2016	Mozambique and Portugal	Children under 5 years and Young adults and adults (15–70 years)	Neither iron deficiency anemia nor nutritional deficiencies were among the ten leading causes of YLLs (with the ratio of observed YLLs to YLLs expected on the basis of SDI in 2015) in Mozambique and Portugal; no specific country findings for anemia were presented.	1990–2015; Estimations of global age-standardised death rates for males versus females, by GBD cause; Estimations of years of lost life(YLLs); social demographic index(SDI) computations	“Hookworm infections can cause iron deficiency anemia, which is then recorded as the underlying cause of death.”
[41]	Forouzanfar et al.	2015	Mozambique and Portugal	Females and Males	Iron deficiency was not among the ten leading risk factors in terms of attributable DALYs for Portugal and Mozambique in 2013 for both sexes combined.	1990–2013; Attributable deaths, years of life lost, years lived with disability, and disability-adjusted life-years (DALYs); estimations of the distribution of exposure	“Iron deficiency accounted for less than 200,000 deaths but was a major cause of DALYs due to its crucial role as a cause of anemia.”
[42]	Vos Theo et al.	2016	Mozambique and Portugal	Children younger that 5 years old and Older children; Young adults (15–39 years old)	Ratio of observed YLDs to YLDs on basis of SDI in 2015 = 0.71 (anemia was among the 10 leading causes of disability in Mozambique). Anemia was not among the 10 leading causes of disability in Portugal.	1990–2015; estimations of anemia prevalence and at different levels of severity; years lived with disability (YLDS) and Social demographic index(SDI) computations	First leading cause in children younger than 5 years and among top 10 causes in older children and adolescents and young adults(15–39 years old)
[43]	Vos Theo et al.	2017	Mozambique and Portugal	Childhood and Women in reproductive age and older women	Ratio of observed YLDs to YLDs on basis of SDI in 2016 = 0.98 (anemia was among the 10 leading causes of disability in Mozambique). Anemia was not among the 10 leading causes of disability in Portugal.	2006–2016; estimations of anemia prevalence; years lived with disability(YLD) and Social demographic index(SDI) computations	Iron deficiency anemia was among the leading level 4 causes of age-standardized YLD for Mozambican females in 2016; iron deficiency anemia was among the main conditions contributing to higher YLD rates in women; In childhood, nutritional deficiencies (mostly iron deficiency anemia) were among the main causes of YLDs.
[45]	Naghavi et al.	2017	Mozambique and Portugal	Children under 5 years and Young adults and adults (15–70 years)	Neither iron deficiency anemia nor nutritional deficiencies were among the ten leading causes of total YLLs (with the ratio of observed YLLs to YLLs expected on the basis of SDI in 2016) in Mozambique and Portugal; no specific country findings for anemia were presented.	2006–2016; estimations of cause-specific deaths and years of life lost (YLLs)	“Age-standardized mortality rates for all nutritional deficiencies decreased by 23·7% (95% UI 15·4–30·8) from 7·26 deaths (6·75–7·86) per 100,000 in 2006 to 5·54 deaths (5·04–6·34) per 100 000 in 2016.”
[46]	Gakidou et al.	2017	Mozambique and Portugal	Pregnant women, Children and Adults	No specific country findings for anemia were presented.	1990–2016; estimations of levels and trends in exposure, attributable deaths, and attributable disability-adjusted life-years (DALYs), by age group, sex, year.	“For deaths, the proportion attributable to measured risk factors, such as communicable, maternal, neonatal, and nutritional causes was 57·9% (55·4–61·0). Risk modification was an important contributor to reductions in communicable, maternal, neonatal, and nutritional causes.”
[47]	Stanaway et al.	2018	Mozambique and Portugal	Children under 5 years and Women of reproductive age	No specific country findings for anemia were presented; globally in 2017, dietary risks were the leading Level 2 risk factor for deaths.	2017; dietary iron deficiency (expressed in terms of prevalence and YLDs); exposure to iron deficiency remained expressed as the counterfactual hemoglobin concentration	-

## Data Availability

Data are contained within the article or Supplementary Materials. Additional information can be requested from the corresponding author upon reasonable request.

## Supplementary Materials

The following supporting information can be downloaded at: https://www.mdpi.com/article/10.3390/ijerph19084685/s1. Supplementary File S1. Methodological quality evaluation of articles included, xls, 01 table. Supplementary File S2. Methodological quality evaluation of articles included, by using STROBE Statement checklist included using STROBE Statement checklist, xls, 01 table, 01 graphic. Supplementary File S2.1. Assessment of the quality of included studies, by all AMSTAR 2 domains, xls, 01 table. Supplementary File S2.2. Assessment of the quality of included studies, by 7 AMSTAR 2 critical domains (%), xls, 02 tables, 01 figure. Supplementary File S3. Description of the articles included, by year of publication, country’s mention, target group, country’s specific findings, period of study, analysis performed, and general findings, xls, 01 table. Additional File S4. Table 1. Search terms and operators used for the identification of publications in the electronic databases, xls, 01 table. Additional File S5. Table 2. Description of the articles included, by year of publication, country’s mention, target group, country’s specific findings, period of study and analysis performed, xls, 01 table. Additional File S6. Description of articles relevant findings by thematic categories, xls, 01 table. Additional File S7. Description of articles identified on the updated search, xls, 01 table. Additional File S8. PRISMA 2020 checklist for systematic review on anemia in pregnant women and children aged 6 to 59 months living in Mozambique and Portugal.

## Abbreviations

CPLP	Community of Portuguese Language Countries
AMSTAR	A Measurement Tool to Assess systematic Reviews
DALYs	Attributable Disadjusted Life Years
EMPIRE	Epidemiological study to determine the prevalence of anemia and iron deficit in the Portuguese population
HELENA	Healthy Lifestyle in Europe by Nutrition in Adolescence
IDA	Iron deficiency anemia
PRISMA	Preferred Reporting Items for Systematic review and Meta-Analysis
PROSPERO	The International Prospective Register of Systematic Reviews
SDI	Social Demographic Index
SDGSs	Sustainable Development Goals
SP	Sulfadoxine-pyrimethamine
STROBE	Strengthening the Reporting of Observational Studies in Epidemiology
UHC	Universal Health Coverage
YLDs	Years Lived with a Disability
